# Worth a Double Take? An In-Depth Review of Lung Retransplantation

**DOI:** 10.3390/jcm12237418

**Published:** 2023-11-30

**Authors:** Gbalekan Dawodu, Shubham Gulati, Helena Bugacov, Daniel Laskey, Brian Housman, Harish Seethamraju, Scott Scheinin

**Affiliations:** 1 Department of Thoracic Surgery, Icahn School of Medicine at Mount Sinai, New York, NY 10029, USA; daniel.laskey@mountsinai.org (D.L.); brian.housman@mountsinai.org (B.H.); scott.scheinin@mountsinai.org (S.S.); 2 Division of Lung Transplantation, Icahn School of Medicine at Mount Sinai, New York, NY 10029, USA; harish.seethamraju@mssm.edu; 3 Icahn School of Medicine at Mount Sinai, 1 Gustave L. Levy Pl, New York, NY 10029, USA; shubham.gulati@icahn.mssm.edu (S.G.); helena.bugacov@icahn.mssm.edu (H.B.); 4 Department of Pulmonary Medicine and Critical Care, Icahn School of Medicine at Mount Sinai, New York, NY 10029, USA

**Keywords:** lung transplantation, lung retransplantation, cardiothoracic surgery, extracorporeal oxygenation, respiratory failure, primary graft dysfunction, chronic allograft dysfunction

## Abstract

Provided advancements in Lung Transplantation (LT) survival, the efficacy of Lung Retransplantation (LRT) has often been debated. Decades of retrospective analyses on thousands of LRT cases provide insight enabling predictive patient criteria for retransplantation. This review used the Preferred Reporting Items for Systematic Reviews and Meta Analyses (PRISMA) guidelines. The PubMed search engine was utilized for articles relating to LRT published through August 2023, and a systematic review was performed using Covidence software version 2.0 (Veritas Health Innovation, Australia). Careful patient selection is vital for successful LRT, and the benefit leans in favor of those in optimal health following their initial transplant. However, the lack of a standardized approach remains apparent. Through an in-depth review, we will address considerations such as chronic lung allograft dysfunction, timing to LRT, surgical and perioperative complexity, and critical ethical concerns that guide the current practice as it relates to this subset of patients for whom LRT is the only therapeutic option available.

## 1. Introduction

In the face of advancements in perioperative management, immunosuppression, and surveillance strategies, we mark improvements in the overall survival of Lung Transplantation (LT) recipients. However, these rates lag when compared to other Solid Organ Transplants (SOT). Lung Retransplantation (LRT) comprises about 5% of total lung transplantations performed globally and has become more frequent in recent years because of the substantial increases in case volume across major centers in the world, as well as the significant improvement in primary LT survival rates [1,2,3].

The efficacy of LRT has often been questioned, yielding decades of retrospective analyses to include thousands of LT and LRT recipients. However, controversies exist regarding its viability and indication. Scrutiny, domestically as well as within the international community, has provided useful insights as to how to best predict and characterize patients who would benefit from LRT. For example, properly categorizing chronic lung allograft dysfunction (CLAD) based on phenotypic expression enables us to predict graft outcomes following LRT [1,2,4]. Much of the relevant literature comprises registry-based analyses, from which it is difficult to account for the outcomes secondary to practices and policies of individual transplant centers and regional regulations. 

This review analyzes the existing literature to establish a foundation for LRT, covering key aspects such as graft dysfunction and indication for retransplantation, patient selection, timing to retransplantation, surgical considerations, ethical concerns, and resource allocation recommendations.

## 2. Materials and Methods

This systematic review was conducted in accordance with the Preferred Reporting Items for Systematic Reviews and Meta Analyses (PRISMA) guidelines. The PubMed web-based search engine was utilized to search for articles relating to LRT published through August 2023. All articles containing mentions of “lung” or “pulmonary” and either “retransplant”, “retransplantation”, “redo transplant”, “redo transplantation”, “repeat transplant”, or “repeat transplantation” in the title or abstract were included in the initial review. The systematic review was registered with PROSPERO (CRD42023465397).

The resulting articles were uploaded to the Covidence systematic review software (Veritas Health Innovation, Melbourne, Australia) version 2.0, and the articles were screened by three independent reviewers. Approval by two reviewers was required for studies to be subsequently considered. Studies that discussed LRT, reviewed literature relating to LRT, discussed ethical considerations of LRT, described surgical approaches for LRT, or limited patients to those undergoing LRT were included. Articles were limited to those published in English and after January 2006, but no restrictions were placed on the study design. Following the decision for inclusion, the resulting articles were classified based on the type of article: commentary, review, or retrospective analysis. These articles were incorporated into this systematic review. 

## 3. Results

**Figure 1:**
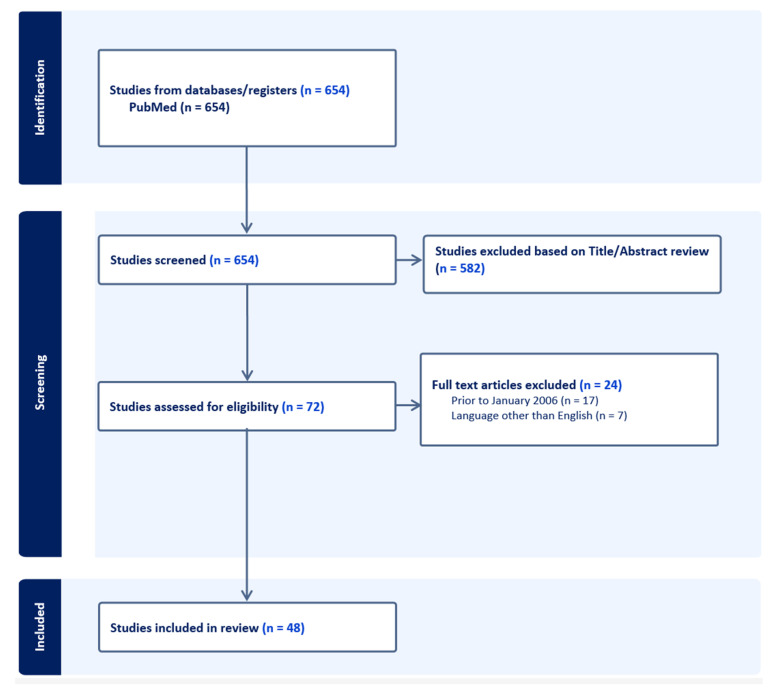
PRISMA flow diagram. PRISMA = preferred reporting items for systematic reviews and meta-analyses.

The search retrieved 654 articles. After excluding duplicates, triplicates, and 582 references based on the title or abstract, 72 full articles were selected. Finally, 48 articles were included in the analysis (Figure 1). 

Table 1 shows the characteristics of studies of lung retransplantation included in the analysis. Table 2 outlines publications that assessed timing to retransplantation. Table 3 outlines publications that assessed the role of extracorporeal membrane oxygenation in LRT. Table 4 outlines publications assessing survival rates with cystic fibrosis in LRT.

## 4. Discussion

Since the first reported lung transplant by Hardy in 1963 [4], indications for LT have broadened over time, spanning a spectrum of diseases of the airways, pulmonary parenchyma, and vasculature. LRT remains the only treatment option for the increasing number of patients who will develop CLAD [2]. 

A 2014 report from the International Society for Heart and Lung Transplantation (ISHLT) indicated a significant increase in LRT cases during the 2005 to 2012 era compared to previous years, albeit associated with worse morbidity and mortality when compared to LT [1]. A more recent study sought to further investigate outcomes in this patient subset [7]. Using an updated ISHLT registry cohort, these authors analyzed 1597 first-time LRT patients between May 2005 and July 2017. Most LRT recipients were between 16 and 64 years old, with 7.5% aged > 65 years. The one-year survival rate of the entire cohort was 73.8%. Survival was similar to Randhawa et al.’s United Network for Organ Sharing (UNOS) Registry-based analysis in the US post-LRT [15], wherein they reported that 1-year survival was lower for LRT recipients in the matched LT cohort (LT 84.8% vs. LRT 76.7%), with higher-volume centers showing superior 1-year survival rates. 

### 4.1. Graft Dysfunction|Chronic Lung Allograft Dysfunction

The indications for LRT differ from primary transplant and most commonly include end-stage CLAD, acute graft failure secondary to primary graft dysfunction (PGD), and irreversible airway complications. Of these, CLAD is most common [1,16,17], with outcomes in this setting historically reported as being superior to other indications of retransplantation [18]. CLAD consists primarily of two phenotypes, bronchiolitis obliterans syndrome (BOS) or its histological counterpart, obliterative bronchiolitis (OB), as well as a restrictive phenotype, restrictive CLAD, previously described as restrictive allograft syndrome (RAS) [19]. One multi-center retrospective study across North America and Europe attempted to stratify outcomes of LRT according to indication by phenotypic expressions of CLAD. These authors found that patients who underwent LRT for BOS had better outcomes than patients for whom the indication for LRT was rCLAD. Restrictive CLAD patients re-developed CLAD earlier and were more likely to re-develop rCLAD [20]. Survival for patients with rCLAD at 1, 3, and 5 years after retransplantation was 59%, 33%, and 28%, with a median graft survival of 1.7 years, whereas patients with BOS experienced survival of 84%, 67%, and 51% with a median graft survival of 5.1 years.

Other recent single institution series comparing survival outcomes for LRT for CLAD to those undergoing LRT for other indications show that despite more complex perioperative courses, survival outcomes may be comparable to patients undergoing LT. The results of these studies are highly subject to selection bias. [21,22]. Despite pre-LRT status, LRT has been shown to be associated with lower 5-year mortality at ~37% [23]. However, outcomes following retransplantation for CLAD appear to be at least partially contingent on the underlying CLAD phenotype.

### 4.2. Patient Selection and Evaluation

When viewed globally, the criteria for candidate selection for lung retransplantation generally mirror those utilized for selection for initial lung transplantation. Prior to the recent implementation of the Composite Allocation Score (CAS) in early 2023, the establishment of the Lung Allocation System (LAS) in 2005 coincided with a rise in the number of LTs performed annually [24]. The objective profiling of patients based on current medical urgency and expected survival measures at one year fashioned a more equitable allocation of donor lungs. Waitlist mortality decreased by nearly half, and median wait time fell from around 2 years to 200 days. Another favorable consequence of this effect was that priority was given to patients who were relisted for transplantation, as the waitlist mortality for CLAD and idiopathic pulmonary fibrosis are similar [16,23,24].

The latter period of the January 1995 to June 2013 era reflects these changes, where 5.1% (799 of 15,631) of single LTs and 3.4% (925 of 27,213) of bilateral LTs reported to the ISHLT were LRTs. The proportion of repeat transplantation, however, remains significantly lower compared to heart, kidney, and liver transplants [1,25]. Despite the increased likelihood of being listed for LRT due to recent changes, the relatively stable number of cases performed may be attributed to specific center decisions that limit retransplantation due to increased surgical complexity [7]. Due to reported poorer outcomes for indications such as Primary Graft Dysfunction (PGD) in the setting of acute graft rejection [20], some institutions have implemented policies to only perform LRT for CLAD indications [21].

Given the nature of the disease, the bulk of the literature regarding LRT is registry-based analyses. An important limitation associated with this approach is the restricted capability to comprehensively analyze the influence of individual transplant center practices on LRT outcomes, as these databases typically lack granular information on center-specific selection criteria and approaches to surgical and medical management following LRT. Therefore, in addition to the complexity of this intervention—both in terms of perioperative approach and in long-term care, the center where the patients undergo evaluation might influence their listing, have an impact on lung allograft acceptance as well as the outcomes of both initial LT and LRT [7,15,26,27].

### 4.3. Timing to Lung Retransplantation|Surgical and Perioperative Considerations

Harhay et al. noted that the majority of LRT occurred > 2 years from the index operation, with the mean interval time to reoperation being 3.5 years [7]. These authors found that 64% had consecutive double lung transplants, and 35.6% had consecutive single lung transplants. The interval between transplants, donor age, and the need for mechanical ventilation preceding LRT were found to be prognostic risk factors for retransplant mortality in the first year, with inter-transplant interval being the strongest factor. Early need for LRT (within 90 days of their LT) had inferior outcomes compared to those needing late LRT. These findings were consistent with Osho et al.’s UNOS Registry-based review [28], which found that in the LAS era, LRT within 90 days of the initial procedure had higher mortality rates, even after adjusting for other factors. Their analysis reported that no significant survival differences were found between repeat and primary transplant recipients after propensity score matching.

When determining the most suitable surgical approach for LRT, several factors must be considered. Among these is the choice between a single or bilateral LRT procedure [29]. In an analysis of survival after LRT, Kon et al. [30], using an SRTR data registry, analyzed 325 patients who had previously undergone SLT. These patients were grouped primarily based on the LRT procedure performed, i.e., ipsilateral LT (ILT), contralateral LT (CLT), and bilateral LT (BLT). Secondarily, patients were grouped based on timing to LRT, defining reoperation within 30 days as “immediate”, 30 to 365 days as “early”, and >1 year as “late”. They reported no significant difference in 30-day, 1-year, and 5-year survival between CLT and BLT (*p* = 0.679). The one-year survival rate of CLT and BLT after SLT appears to be approaching initial LT. However, recipients of both CLT and BLT exhibited a significant survival advantage compared with ILT (*p* = 0.007 and *p* = 0.040, respectively). It is important to reiterate that retrospective registry analysis may not be granular enough to account for many clinical factors that influence patient-level decision choices regarding the choice of reoperation after SLT. Although ILT was associated with increased mortality after initial SLT, this procedure may nevertheless be the best option under some circumstances, such as severe, persistent PGD. Upon multivariate analysis, the authors of this study found that PGD as the diagnostic indication for LRT did not by itself account for the higher risk of 6-month mortality after ILT compared with CLT and BLT.

Schummer et al. [31] sought to answer the question of whether survival varied between single and double LRT in comparison to the initial procedure. Using UNOS data during the Lung Allocation Score (LAS) period, patients were divided into four groups based on first followed by second transplant type, respectively: single then single (S-S), double then single (D-S), double then double (D-D), and single then double (S-D). They reported worse survival for recipients of single LRT when compared to recipients of double LRT; however, when stratified by consecutive transplant type (single versus double), they found no difference in survival among the four study groups. Although the study groups were similar in the proportion of gender, waitlist time, and oxygen requirement at the time of retransplant, they were dissimilar in age, BMI, creatinine, time to retransplant, LAS, and diagnosis. This is likely a reflection of the method of stratification, as a patient with chronic obstructive pulmonary disease is more likely to be older and receive a single lung, whereas a patient with CF is more likely to be younger, have a lower BMI, and receive a double lung transplant. The authors bring up an essential point, especially in the face of a limited supply of donor lungs in the face of increasing demand, suggesting that LRT with single lungs given the right patient characteristics may be appropriate to optimize the use of donor lungs.

Regarding stays in the Intensive Care Unit (ICU), a report by Halloran et al. found that patients who underwent LRT had extended periods of mechanical ventilation and, as a result, longer stays in the ICU when compared to those who had primary lung transplantation [22]. This finding, however, is in contrast with several other reports, which found no difference in the duration of postoperative mechanical ventilation for both primary transplant and retransplant patients [21,32].

The surgical approach for LRT is largely described as being performed via Thoracosternotomy or clamshell (CS) incision. However, the CS incision is not without significant morbidity, including sternal wound dehiscence, sternal instability, and wound infection [33]. Some authors have described implementing less invasive strategies aimed at mitigating morbidity and improving early outcomes. Sommer et al. [17], via a single institution study, analyzed 87 LRT patients, dividing the cohort into groups based on the era during which LRT was performed. A less invasive protocol, off-pump, sternal-sparing Bilateral Anterior Thoracotomies (BAT), was applied to patients who underwent LRT between April 2010 and March 2016. Despite a longer mean operation time in the less invasive era (406.1 ± 86.8 vs. 296.1 ± 96.1, *p* < 0.0001), the authors reported several advantages over those who had the procedure before April 2010. Patients had better survival rates both at 30 days (98% vs. 76.3%) and one year (80.6% vs. 63.2%) after LRT. There was a significant decrease in the need for dialysis due to acute renal failure (10.2% vs. 39.5%) in the group that received less invasive techniques. Patients in the more recent era had shorter times until extubation (1 day vs. 11.5 days) and shorter stays in the Intensive Care Unit (ICU) (4 days vs. 12.5 days).

Bhama et al. [34], in a retrospective single-institution review, described the use of sternal-sparing BAT in 15 consecutive double LRT patients. Although they noted no significant difference in the lengths of intensive care unit (ICU) and total hospital stay between the groups, patients who underwent LRT via BAT had a non-significant trend towards a shorter stay compared with the CS group. The authors did not account for operating time. One significant technical challenge they described relates to the dissection of the pulmonary artery from the bronchus, which are often densely adhered to each other, which they accounted for by obtaining intrapericardial control of the main pulmonary artery to maintain vascular control during this dissection. Despite excellent exposure to the hilar structures, exposure to the diaphragmatic and apical surfaces is certainly suboptimal compared with the CS approach, making dissection in those areas more challenging, especially in the setting of dense adhesions. [34,35]. We believe that the choice of approach for this procedure should depend on surgeon–institutional experience, as well as individualized patient factors.

With regard to induction therapy in this patient subset, we realize that strategies implored vary according to institutional practice. As with ours, attained immunotolerance in a patient for which LRT is indicated will not necessitate the receipt of induction therapy. However, for example, in patients with CLAD in the setting of recurrent acute rejection episodes, induction therapy is routinely used.

### 4.4. Lung Retransplantation in Cystic Fibrosis

Survival in cystic fibrosis (CF) has improved dramatically over the last several decades, in part due to the implementation of CF specialty centers, as well as the utilization of modular therapy targeting the underlying protein defect. However, the primary source of morbidity and mortality remains pulmonary disease, with CF comprising 23.4% and 15.8% of all bilateral lung transplants and total lung transplants worldwide, respectively, at a mean age of 27.8 years [14,36,37,38]. Despite better outcomes after LT, CF patients seem to lose this survival benefit at the time of LRT as their outcomes are relatively similar to other diagnoses, based on reports by the ISHLT Registry. One single institution retrospective review reported comparable findings regarding the equilibration of outcomes following double LRT in this patient subset [1,39]. A recent UNOS registry-based analysis reported an overall CF cohort with significantly worse 5-year graft survival (47.9%) following double LRT when compared to the CF initial LT. While these findings may appear superior to the 37% reported by the ISHLT report, it is important to note that the latter cohort included a much older cohort (all LRT performed between January 1995 and June 2012) compared to their LAS-era group. The only predictor of graft failure at 3 years noted was the use of mechanical ventilatory support [1,14]. Alshehri et al. postulate that given a decrease in the number of LT for CF since the implementation of ETI, as well as non-indication for ETI in chronic rejection, as post-LT lungs no longer have CF [12], the amount of LRT performed for CF will outweigh primary LT in the near future.

### 4.5. ECMO in Lung Retransplantation

Despite the implementation of contemporary accepted strategies, the use of intubation and mechanical ventilation (IMV) continues to be linked with reduced overall survival following LT [40,41,42]. The increasing efficacy and safety of the use of extracorporeal membrane oxygenation (ECMO) as a bridge to LT, along with the improvements in contemporary results facilitated by enhanced technology and the expanding expertise in this domain, have established ECMO as a valid therapeutic option for individuals with advanced pulmonary disease [43,44,45].

Using the SRTR database, Hayanga et al. [40] analyzed 854 LRT patients of whom 6.8% underwent the use of ECMO support prior to LRT. Primarily assessing survival outcomes, the authors reported significant differences in 30-day (67.3% vs. 91.2%, *p* = 0.0002), 90-day (67.3% vs. 91.2%, *p* = 0.0002), 1-year (44.8% vs. 69.3%, *p* = 0.0006) and 5-year (21.4% vs. 38.1%, *p* = 0.02) survival between ECMO and non-ECMO groups. A recent UNOS Registry study investigated the outcome after 33 ECMO-bridged retransplants and found a significantly decreased survival rate after ECMO-bridged retransplants compared to first transplants with bridging ECMO or retransplants without [11].

In a recent study conducted by the Vienna group, the outcomes of retransplants were assessed, specifically in relation to various extracorporeal life support methods [46]. The findings indicated that postoperative mortality and 2-year survival rates were 20% and 53% for patients without any bridging support (*n* = 23), 29% and 29% for those with bridging support involving invasive mechanical ventilation (IMV) with or without extracorporeal membrane oxygenation (ECMO) (*n* = 11), and 0% and 60% for patients with bridging support involving awake ECMO (*n* =  5). These authors inferred that LRT involving awake ECMO as a bridging strategy exhibited outcomes comparable to non-bridged elective retransplants.

### 4.6. Ethical Considerations and Patients’ Perspectives

Allocation of donor lungs, which remain a scarce resource, implicates ethical issues of social justice, non-maleficence, and benevolence in the framework of LRT [47,48]. Andrews et al. invoked the notion of social justice as they asked the question, “is it fair that someone is given 2 and often more transplantations when others have been waiting for up to more than 5 years for their first organ?” [47]. Then again, as posed by Kawut et al., is it justifiable to prioritize initial LT over LRT for someone with a failed graft, potentially saving more years of life? In no effort to overly simplify this ethical dilemma, it is safe to say that the ethical framework for retransplantation lags behind its successful implementation. A key element to the fair allocation of organs for retransplantation lies in the accuracy of the metric of “net benefit” used to distribute organs [16].

In addressing this ethical dilemma, the OPTN acknowledges that indications for repeat transplantation may occur any time after transplantation and, for many reasons, many beyond the control of the patient, stating that repeat transplantation should not be the sole criterion either restricting or promoting candidacy [49]. While a universally accepted ethical allocation algorithm for retransplantation remains elusive, substantial endeavors have been dedicated to augmenting organ availability and outlining the defining characteristics of an optimal candidate for LRT to tackle this challenge. Careful patient selection accounts for an important part of the success of LT, and in view of LRT, the screening process must be even more strictly performed in favor of the proportion of patients for whom it will be beneficial without compromising patients who are waiting for their first transplantation [50].

A recent study aimed to describe the experiences of patients and their next of kin (NoK) while awaiting LRT, focusing on a small group with special circumstances and needs [51]. Via qualitative content analysis, data from interviews with seven adult patients and seven NoK from a regional lung transplantation were analyzed. Patients’ thoughts ranged from both a medical and existential perspective. Several patients pondered the interference of higher powers testing them, and some patients expressed gratitude for the years they had received after the first transplant. As much as these patients expressed great hopefulness about having an active life again, thoughts of their mortality loomed in the background. The precise percentage of patients who may not qualify for or subsequently be deemed unsuitable candidates for SOT is uncertain. It is quite plausible to speculate that this figure is likely even smaller for individuals requiring repeat transplantation, underscoring the significance of incorporating supportive care, including palliative care, within the care plan for patients undergoing evaluation for or awaiting LRT.

## 5. Conclusions

There continues to be a subset of patients for whom LRT is not only indicated but remains the only therapeutic option in end-stage lung disease. Improved outcomes witnessed in the preceding decades have been largely attributed to technological advancements, improvements in surgical and perioperative approaches, and refinements in immunosuppression and surveillance strategies.

Thoughtful patient selection is vital for successful lung retransplantation due to the intricate surgical and perioperative challenges. Patients who benefit the most are those with good survival after their initial transplant. Current literature is not without variability to definitions and inferences; in large, from a predominantly retrospective pool. We postulate that the implementation of consensus guidelines will be instrumental in standardizing the approach to managing this complex patient subset with an already challenging endeavor, setting the framework for further research.

## Figures and Tables

**Table 1:** Characteristics of studies of lung retransplantation (LRT) included in the analysis.

	All Studies
Range, mean journal impact factor	0.49–51, 5.75
Geographical origin, *n* (%)	
North America	32 (66.67%)
Asia	2 (4.16%)
Europe	14 (29.16%)
Randomized control trials	
Single center, *n* (%)	15 (31.25%)
Multi-center, *n* (%)	4 (8.33%)
Registry-based analyses, *n* (%)	16 (33.33%)
Reviews, *n* (%)	9 (18.75%)

**Table 2:** Timing to lung retransplantation (LRT).

Title	Study Type	Sample Size	Study Period	Findings|Inference	Reference, Year
Time since primary transplant and poor functional status predict survival after redo lung transplant.	Retrospective, SRTR * registry	739 LRT	2005–2019	Functional status and time from primary lung transplant are important predictors for worse outcomes.	Aggarwal, 2022 [5]
Thoracic retransplantation: Does time to retransplantation matter?	Retrospective, UNOS/OPTN ª registry	1779 (871 LRT)	2005–2020	A shorter time to LRT for lung is associated with decreased survival.	Ganapathi, 2022 [6]
Epidemiology, risk factors, and outcomes of lung retransplantation: An analysis of the International Society for Heart and Lung Transplantation Thoracic Transplant Registry.	Retrospective, ISHLT º registry	1597 LRT	2005–2017	The majority of LRTs were 2 or more years after their initial transplant (34.3% within 2 ≤ 5 years, and 33.8% ≥ 5 years).	Harhay, 2022 [7]

* Scientific Registry of Transplant Recipients. ª United Network for Organ Sharing/Organ Procurement and Transplantation Network. º International Society for Heart and Lung Transplantation.

**Table 3:** Role of extracorporeal membrane oxygenation (ECMO) in lung retransplantation (LRT).

Title	Study Type	Sample Size (LRT Recipients)	Study Period	Findings|Inference	Reference, Year
Extracorporeal life support as a bridge to pulmonary retransplantation: prognostic factors for survival in a multicentre cohort analysis.	Retrospective, multi-center	5219 (260)	2005–2019	ECLS ° as a bridge to LRT was an independent risk factor for mortality in the short-term to long-term follow-up.	Inci, 2022 [8]
Outcome after extracorporeal membrane oxygenation-bridged lung retransplants: a single-centre experience.	Retrospective, single institution.	230 (11)	2004–2016	OS ∞ was significantly lower with bridging prior to LRT, with a lower rate of CLAD º -free survival.	Abdelnour-Berchtold, 2019 [2]
Extracorporeal Life Support as Bridge to Lung Retransplantation: A Multicenter Pooled Data Analysis.	Retrospective, multi-center pooled.	17	2000–2014	Awake ECMO ^‡^ patients with an inter-transplant interval of >2 years had 1-year survival approaching primary lung transplantation.	Collaud, 2016 [9]
Extracorporeal membrane oxygenation as a bridge to lung re-transplantation: Is there a role?	Retrospective, SRTR *, and UNOS/OPTN ª registries.	854	1988–2012	1-year (44.8% vs. 69.3) and 5-year (21.4% vs. 38.1%) survival rates were each significantly lower in the ECMO group compared with the non-ECMO group.	Hayanga, 2016 [10]
Extracorporeal membrane oxygenation and retransplantation in lung transplantation: an analysis of the UNOS registry.	Retrospective, UNOS/OPTN ª registry	15,772 (581)	2001–2012	Significant survival differences between LT with and without ECMO vs. with and without ECMO.	Hayes, 2014 [11]

∞ Overall survival. ^‡^ Extracorporeal membrane oxygenation. º Chronic Lung Allograft Dysfunction. * Scientific Registry of Transplant Recipients. ª United Network for Organ Sharing/Organ Procurement and Transplantation Network. ° Extracorporeal life support.

**Table 4:** Cystic Fibrosis (CF) outcome in lung retransplantation (LRT).

Title	Study Type	Sample Size	Study Period	Findings|Inference	Reference, Year
Cystic fibrosis survival outcomes following second lung transplant: The north American experience.	Retrospective, multiple registries. ª	1226 (395 LRT)	1984–2019	Lower 1-, 3- and 5-year survival following LRT vs. LT. Less than 5% of the pediatric population.	Alshehri, 2023 [12]
Perioperative Outcomes During Double-Lung Transplantation and Retransplantation in Cystic Fibrosis Patients: A Monocentric Cohort Study.	Retrospective, single-institution	282 (24 LRT)	2012–2021	No significant difference was noted in perioperative outcomes between Double LRT and LT; however, higher prophylactic use of pre-op ECMO ∞.	Fessler, 2023 [13]
Outcomes following lung re-transplantation in patients with cystic fibrosis.	Retrospective, UNOS/OPTN º registry	277 Double LRT	2005–2020	LRT was more common in the adult population. Higher LAS ^‡^ at listing, as well as >increase in scoring from listing to LRT. 7.6% (vs. 4.0%, *p* < 0.003) bridged using ECMO ∞.	Chan, 2022 [14]

ª Organ Procurement and Transplantation Network (OPTN), US CF Foundation (CFF) Patient Registry (US CFFPR), and the Canadian CF Registry (CCFR). º United Network for Organ Sharing/Organ Procurement and Transplantation Network. ^‡^ Lung Allocation Score. ∞ Extracorporeal membrane oxygenation.

## Data Availability

No new data were created or analyzed in this study. Data sharing is not applicable to this article.
